# The Cholesterol-Dependent Cytolysin Signature Motif: A Critical Element in the Allosteric Pathway that Couples Membrane Binding to Pore Assembly

**DOI:** 10.1371/journal.ppat.1002787

**Published:** 2012-07-05

**Authors:** Kelley J. Dowd, Rodney K. Tweten

**Affiliations:** Department of Microbiology and Immunology, The University of Oklahoma Health Sciences Center, Oklahoma City, Oklahoma, United States of America; The University of Texas-Houston Medical School, United States of America

## Abstract

The cholesterol-dependent cytolysins (CDCs) constitute a family of pore-forming toxins that contribute to the pathogenesis of a large number of Gram-positive bacterial pathogens.The most highly conserved region in the primary structure of the CDCs is the signature undecapeptide sequence (ECTGLAWEWWR). The CDC pore forming mechanism is highly sensitive to changes in its structure, yet its contribution to the molecular mechanism of the CDCs has remained enigmatic. Using a combination of fluorescence spectroscopic methods we provide evidence that shows the undecapeptide motif of the archetype CDC, perfringolysin O (PFO), is a key structural element in the allosteric coupling of the cholesterol-mediated membrane binding in domain 4 (D4) to distal structural changes in domain 3 (D3) that are required for the formation of the oligomeric pore complex. Loss of the undecapeptide function prevents all measurable D3 structural transitions, the intermolecular interaction of membrane bound monomers and the assembly of the oligomeric pore complex. We further show that this pathway does not exist in intermedilysin (ILY), a CDC that exhibits a divergent undecapeptide and that has evolved to use human CD59 rather than cholesterol as its receptor. These studies show for the first time that the undecapeptide of the cholesterol-binding CDCs forms a critical element of the allosteric pathway that controls the assembly of the pore complex.

## Author Summary

The CDCs are a large family of pathogenesis-associated pore-forming toxins that are expressed by many Gram-positive pathogens. The conserved undecapeptide motif of the CDCs has been regarded as the signature peptide sequence for these toxins, yet its function has remained obscure. The studies herein show that the undecapeptide forms a critical structural element in the allosteric pathway that couples membrane binding to cholesterol to the initiation of distal structural changes, which are required for the assembly of the pore forming complex. These studies provide the first insight into the function of this highly conserved sequence and show that through evolution this pathway is missing in the CD59-binding CDCs.

## Introduction

**Figure 1:**
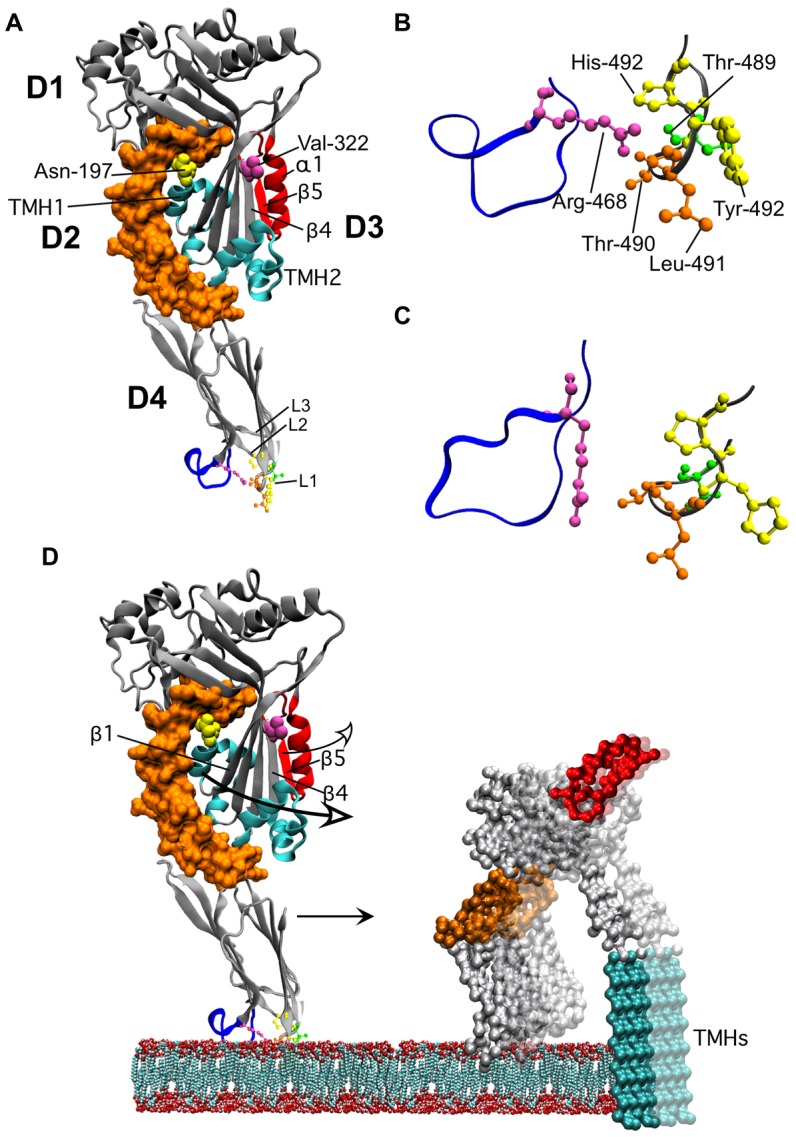
The molecular structures of PFO and ILY. Shown in A is a ribbon representation of the crystal structure of PFO [7]. The domain 3 β5 strand and associated α-helix (α1) that swing away from β4 are highlighted in red. The locations of Asn-197 (at the D2–D3 interface) and Val-322 (buried under the loop formed by α1β5) are shown in space-filled atoms. The twin α-helical bundles in D3 (cyan) extend into the twin transmembrane β-strands (TMHs). The conserved undecapeptide is shown in blue in D4. In panel B an enlarged view of the conserved undecapeptide loop and the CRM containing loop L1 of PFO is shown. In Panel C the analogous structures are shown for ILY as are shown in panel B for PFO.All structures were derived from the crystal structures of PFO and ILY [6], [7]). In panel D we show the structural changes in PFO as it makes the transition from the bound monomer state to the membrane embedded oligomer. The membrane embedded monomer structure is based on the 3D reconstruction of the pneumolysin pore fitted with the PFO crystal structure [57]. D3 breaks its contacts with D2 and swings out in order to extend the α-helical bundles (in cyan) into the twin TMHs. This transition also repositions Asn-197 from the D2–D3 interface to a solvent exposed position within the lumen of the membrane pore. Prior to or simultaneously with the disruption of the D2–D3 interface the α1β5 loop (red) swings away from β4 thus exposing the edge β4 (as well as exposing Val-322 to the solvent), which can then pair with β1 of a second monomer. Upon transition to the pore the oligomeric complex undergoes a 40 Å vertical collapse to insert the β-barrel pore into the bilayer [23]. After β5 breaks contact with β4 the location of α1β5 loop is not known, its position in the model is for illustrative purposes only. All structures were generated using VMD [58].

The cholesterol-dependent-cytolysin (CDC) family of toxins consists of over 25 members that are produced by many different species of Gram-positive bacterial pathogens [1] and contribute in various ways to the pathogenesis of these organisms [2], [3], [4], [5]. Members of this family exhibit high levels of homology in their primary structures (40–70%) and in the crystal structures of their soluble monomers [6], [7], [8], [9]. The region within the CDC primary structure that exhibits the highest degree of sequence identity is an 11-residue peptide known as the undecapeptide or tryptophan-rich motif, which is located near the C-terminus of the molecule in domain 4 (D4) (Fig. 1). The undecapeptide (ECTGLAWEWWR) is the signature motif for the CDCs [1] and so proteins exhibiting this peptide sequence have a high probability of belonging to the CDC family. The pore forming mechanism of the CDCs that use cholesterol as their receptoris highly sensitive to changes in the primary structure of the undecapeptide [6], [10], [11], [12], [13], [14], [15]. These studies suggest that the undecapeptide plays an important role in the CDC pore-forming mechanism, yet since Iwamoto et al. [16] began studying the effects of chemically altering the undecapeptide in 1987 its contribution to the pore forming mechanism of the CDCs has remained elusive.

The undecapeptide is located at the tip of D4 of the CDC structure, as shown in the structure of the CDC produced by *Clostridium perfringens*, perfringolysin O (PFO) (Fig. 1). D4 also contains the cholesterol recognition/binding motif (CRM) and two other short loops (L2 and L3) near the undecapeptide (reviewed in [17]). Upon recognition of membrane cholesterol by the CRM,loops L2 and L3 insert into the membrane. These interactions anchor the monomers in a perpendicular orientationto the membrane surface where the tip of D4 is anchored to the membrane surface and the top of D3 resides about 113 Å above the membrane surface [18], [19], [20]. Although the sidechains of several residues of loops L2 and L3 and the undecapeptide insert into and anchor the monomers to the membrane they do not penetrate deeply into the bilayer core [19], [21].

It had been generally accepted in the field that the undecapeptide motif wasthe CRM of the CDCs, although this function had never been demonstrated unambiguously. An early study by Iwamoto et al. [16] showed that chemical modification of the undecapeptide cysteine caused independent defects in both binding and pore formation. Since that time it has been shown that mutation of many of the undecapeptide residues often affects both binding and pore formation [6], [10], [11], [12], [13], [14], [15]. We recently showed, however, that the CRMresides in the nearby D4 loop L1 (Fig. 1) and is comprised of a threonine-leucine pair that is strictly conserved in all known CDCs [22]. Upon cholesterol binding by the CRM the nearby loops L2 and L3 and the conserved undecapeptide insert into the bilayer surface and anchor the monomer in a perpendicular orientation to the membrane surface [19], [21], [23], [24]. Membrane binding in conjunction with monomer-monomer interactions [20] initiates and drives a dramatic series of secondary and tertiary structural changes in D3, which is about 60 Å distant from the tip of D4 (Fig. 1). These structural changes are necessary for the assembly of the membrane bound monomers into the large oligomeric pore complex [23], [24], [25], [26], [27]. Soluble monomers of PFO do not exhibit these D3 structural changes, even at the high concentrations required for crystallization of the protein [28]: membrane binding is required to initiate the structural changes in D3 [20], [25].

As indicated above, the pore-forming mechanism of PFO-like CDCs is highly sensitive to mutations in the undecapeptide [6], [10], [11], [12], [13], [14], [15]. Furthermore, the conformational changes in the PFO undecapeptide, reflected by the membrane insertion of its tryptophan residues, are conformationally coupled to the structural changes in TMH1 required for the formation of the β-barrel pore [20]. This observation suggests that the undecapeptide of PFO is involved in the allosteric coupling of membrane binding to the initiation of the D3 structural changes that are necessary for monomer-monomer interaction and the formation of the oligomeric β-barrel pore complex.

A small family of CDCs, typified by *Streptococcus intermedius* intermedilysin (ILY) use human CD59 as their receptor, rather than cholesterol [29], [30], [31]. The D3 structural changes in ILY can be initiated by binding to human CD59 in membranes that are largely, though not completely depleted of cholesterol [32]. ILY still requires a CRM-mediated membrane interaction with cholesterol to maintain its anchor to the membrane surface (it disengages from CD59 during prepore to pore conversion [22], [33]), but it remains unclear if cholesterol binding also participates in initiation of the D3 structural changes necessary for assembly of the oligomer pore complex. Interestingly, in contrast to the CDCs that use cholesterol as their receptor, the pore forming mechanism of ILY is comparatively insensitive to mutations within its undecapeptide [6], which suggests that it may not play as significant of a role in the pore forming mechanism of these toxins.

In the present study we performed a detailed molecular analysis of a point mutation in the undecapeptide of PFO that reduces its pore-forming activity 100-fold, whereasthe analogous mutation has no significant effect on the mechanism of ILY [6]. In PFO this mutant blocks all measurable structural transitions in D3 and prevents the stable interaction of membrane-bound monomers. We further show that the effect of this mutation on the activity of PFO is similar to that observed for cholesterol bound native ILY in the absence of CD59. These results show that the undecapeptide of PFO is a critical structure within the allosteric pathway of PFO that couples cholesterol binding to the initiation of structural changes within D3, which lead to the formation of the β-barrel pore. We further show that this pathway appears to be missing in the CD59-binding ILY, so that assembly of its pore complex is initiated by its interaction with CD59 rather than cholesterol.

## Results

### Cytolytic activity of PFO mutated at Arg-468

Arg-468 is the last residue of the PFO undecapeptide (ECTGLAWEWW**R**), as well as in the ILY undecapeptide (GATGLAWEPW**R**). Substitution of the PFO undecapeptide at this residue with alanine decreases its hemolytic activity 100-fold (Table 1), whereas substitution of the analogous residue in ILY has little effect on the activity [6]. A series of mutants were generated for Arg-468 of PFO to examine the effects of size, length and charge of the residue atposition 468 on the hemolytic activity of PFO (Table 1). Neither conservative nor non-conservative substitutions were tolerated: all substitutions decreased hemolytic activity ≥100-fold. Based on the crystal structure of PFO the only intramolecular contacts established by Arg-468 are hydrogen bonds between its sidechain NH1 and the CRM carbonyls (Fig. 1B). Interestingly, this contact is lost in the ILY monomer (Fig. 1C), which presumably results from differences in its undecapeptide structure [6]. We selected the PFO^R468A^ mutant for further studies into the defect(s) induced by substitution of the Arg-468 residue on the PFO pore-forming mechanism.

**Table 1:** Cytolytic activity of PFO derivatives with mutations in the Arg-468 residue.

Toxin	EC_50_ (M)	% WT
PFO	6.0×10^−11^	100
R468A	6.0×10^−9^	1
R468K	5.0×10^−9^	1
R468Q	2.4×10^−8^	<1
R468E	3.9×10^−8^	<1

Cytolytic activity of PFO and Arg-468 mutants is shown as the effective concentration (EC_50_) of toxin required for 50% lysis of human erythrocytes under standard assay conditions (see Materials and Methods for details).

### PFO^R468A^ membrane binding

We have shown that a conserved Thr-Leu pair in Loop 1, and not the undecapeptide, is responsible for CDC binding to membrane cholesterol [22], yet mutations within the conserved undecapeptide were often observed to affect binding [6], [13], [34]. To confirm that the loss of hemolytic activity by the PFO^R468A^ mutant was not due solely to a defect in binding we examined the ability of the mutant to bind to human RBCs by flow cytometry. In order to prevent cell lysis at high concentrations of toxin, derivatives of native PFO and PFO^R468A^ were generatedin which an engineered disulfide was introduced between residuesThr-319 in β4 and Val-334 in β5 that prevent the rotation of β5 away from β4 in domain 3 (PFO^β4β5^ and PFO^R468A•β4β5^). The engineered disulfide thereforepreventsthe formation of a functional pore by blocking the intermolecular interaction of β1 of one monomer with β4 of another monomer [25]. A third cysteine was substituted at residue Asp-30 in both mutants, which is at the amino terminus of PFO, so that specific fluorescent probes could be introduced into these mutants. This mutation does not affectthe structure of PFO or its function [35].

**Figure 2:**
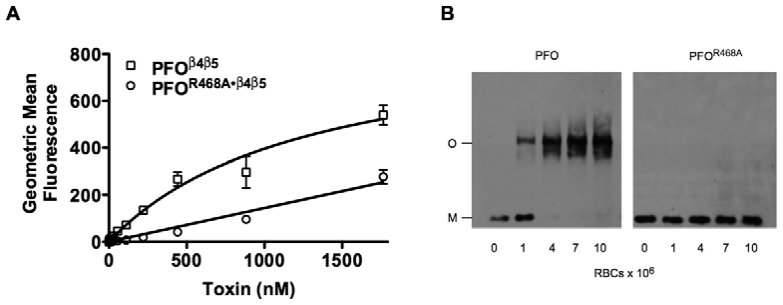
Binding and oligomerization of PFO and PFO^R468A^. (A) Binding of PFO^β4β5^ and PFO^R468A•β4β5^ to human RBCs (4×10^6^/ml in a final volume of 0.5 ml) was measured by flow cytometry. The disulfide locked β4β5 versions of each protein [25] were used to prevent the lysis of the RBCs during flow cytometry. (B). Oligomerization of PFO and PFO^R468A^(both toxins were maintained at 440 nM)on human RBCs (concentrations ranged from 2.5×10^7^/ml to 2.5×10^8^/ml in a final volume of 40 µl) was determined using SDS-agarose gel electrophoresis (SDS-AGE) and the proteins were detected with anti-PFO antibody after transfer to nitrocellulose paper. The analyses are representative of at least 3 experiments.

At the highest concentration of PFO^R468A^we observed about a 50% decrease in binding to hRBCs compared to PFO, although at lower concentrations this difference was greater (Fig. 2A). The decrease in binding, however, doesn't account for the 100-fold decrease in cytolytic activity. PFO follows an ordered series of coupled conformational changes that are initiated by binding [19], [20], [23], [25], therefore the major defect induced by the PFO^R468A^ mutation affects an event after binding, which then prevents formation of the pore complex.

### Oligomerization of PFO^R468A^

Upon membrane binding PFO monomers oligomerize into large SDS-resistant prepore complexes containing approximately 36 monomers [23]. An oligomerization assay was performed with the PFO^R468A^ mutant. Due to the lower binding affinity observed in Fig. 2A we increased the concentration of human red blood cells (hRBCs) to ensure complete binding of PFO^R468A^. The concentration of hRBCs in the binding studies in Fig. 2A was maintained at 4×10^6^/ml whereas in the oligomerization assay shown in Fig. 2B the concentration hRBCs ranged from 2.5×10^7^ to 2.5×10^8^/ml, which wasapproximately 6–60 fold higher than the concentration used in the flow cytometry assay. After the toxins were allowed to bind, the samples were solubilized with SDS without heating and separated by SDS-agarose gel electrophoresis (SDS-AGE), which separates the monomer and oligomer forms [36]. Native PFO readily formed SDS-resistant oligomers at all concentrations of RBCs (Fig. 2B) whereas PFO^R468A^did not form detectable levels of SDS-resistant oligomers (Fig. 2B). Therefore, the major defect in the PFO^R468A^ pore-forming mechanism follows binding and prevents the formation of an SDS-stable oligomer.

### Rotation of β5 away from β4

Several structural transitions in domain 3 are initiated by membrane binding, which are required for oligomerization and pore formation [25], [26], [27]. One of these structural transitions is the rotation of β-strand 5 (β5) away from the adjacent β-strand 4 (β4) of the core β-sheet in domain 3 (Fig. 1A), whichcontributes to the formation of the SDS-resistant prepore oligomer [25]. Rotation of β5 away from β4 allows the formation of edge-on hydrogen bonds between the peptide backbones of β4 and β1 of two membrane-bound monomers [25].

**Figure 3:**
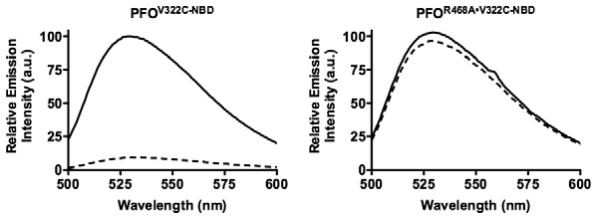
Disruption of the β4β5 interface of PFO and PFO^R468A^. A cysteine was substituted for Val-322, located in the D3 β4 strand. Each derivative was labeled with NBD and incubated in the presence (dashed line) and absence (solid line) of human erythrocyte ghost membranes. The fluorescence emission intensity of NBD was measured from 500–600 nm. The data are representative of 3 experiments.

The disruption of the β4/β5 interaction can be followed spectroscopically using the environmentally sensitive fluorescent probe, NBD (7-nitrobenz-2-oza-1,3 diazole) [27], which is positioned on the sulfhydryl of a cysteine substituted for Val-322 in β4 (Fig. 1A). Val-322 is buried under the residues of β5 and so a probe positioned here is in a hydrophobic pocket. The fluorescence emission of NBD is quenched by water, therefore as β5 rotates away from β4 the NBD positioned in β4 moves from a nonpolar to polar environment, which results in a decrease in its fluorescence emission intensity as it is exposed to the aqueous milieu [25]. The PFO^R468A•V322C-NBD^ mutant exhibited virtually no change in the NBD emission compared to functional PFO^V322C-NBD^ (Fig. 3). These results show that the rotation of β5 away from β4 does not occur in membrane bound PFO^R468A^.

### Monomer-monomer interaction of PFO^R468A^

The studies above show that PFO^R468A^does not form SDS-resistant oligomers, which is likely due to the loss of the intermolecular β1–β4 interaction of monomers. This observation, however, did not rule out the possibility that PFO^R468A^monomers could still form a SDS-sensitive oligomer. To determine whether PFO^R468A^formed SDS-sensitive oligomers the PFO^R468A^ monomer association was examined using fluorescence resonance energy transfer (FRET). A cysteine was substituted for the amino terminal Asp-30 and labeled with either donor fluorophore (D) (Alexa Fluor 488) or acceptor fluorophore (A) (Alexa Fluor 568). A mixture containing a 4∶1 molar ratio of A-labeled PFO^R468A^ or unlabeled PFO^R468A^(U) to D-labeled toxin was incubated with membranes and fluorescence emission intensity of D was measured.

**Figure 4:**
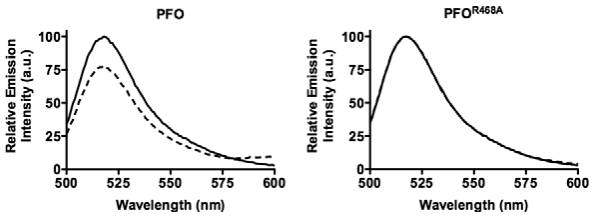
FRET-detected monomer association of PFO and PFO^R468A^. A cysteine was substituted for Asp-30, located in domain 1 and the derivatives were labeled with Alexa Fluor 488 (donor, D) or Alexa Fluor 568 (acceptor, A).A 4∶1 molar ratio of A-labeled PFO^R468A^ (dashed line) or unlabeled PFO^R468A^ (U; solid line) to D-labeled toxin was incubated in the presence of human erythrocyte ghost membranes and fluorescence emission intensity of D was measured.

When membrane-bound PFO monomers associate to form the prepore oligomer the distance (R_0_) between D and Afluorescent dyes on the monomers decreases, which results in the FRET-dependent quenching of the D emission (R_0_ is typically<10 nm) [37]. As expected, we observed an A-dependent quenching of the D emission for functional PFO as it oligomerized [35], [37], whereas no change in the donor fluorescence was observed for PFO^R468A^(Fig. 4).This result shows that the PFO^R468A^ monomers do not interact, or only form transient interactions that cannot be detected by FRET. FRET requires the donor and acceptor pair be at a fixed distance during the lifetime of the donor emission, which for Alexa-488 is approximately 4 ns [38]. Therefore the PFO^R468A^ monomers are, at most, only interacting briefly within a timeframe that is shorter than the fluorescence lifetime of the Alexa dye.

### The status of the domain 3 TMHs in PFO^R468A^

The D2–D3 interface is disrupted in order to extend the D3 α-helical bundle into transmembrane β-hairpin 1 (TMH1) [27], which together with TMH2 ultimately contribute to the formation of the membrane spanning β-barrel pore [26], [27]. First, the α-helical bundle that forms TMH1 must break its interaction with D2 to unravel and form the extended β-hairpin structure, which eventually inserts into the bilayer as part of the β-barrel pore [27]. Disruption of the TMH1 contact with D2 can be measured by placing a NBD probe on a cysteine substituted for Asn-197inTMH1 (Fig. 1). Asn-197 resides at this interface and undergoes a nonpolar to polar transition as the α-helical bundle breaks contact with D2 and unravels to form the extended β-hairpin [27]. The subsequent insertion of the β-barrel pore can be followed by placing a NBD probe on cysteine-substituted Ala-215 in TMH1, which undergoes a polar to nonpolar transition as its sidechain inserts into the bilayer core [27].

**Figure 5:**
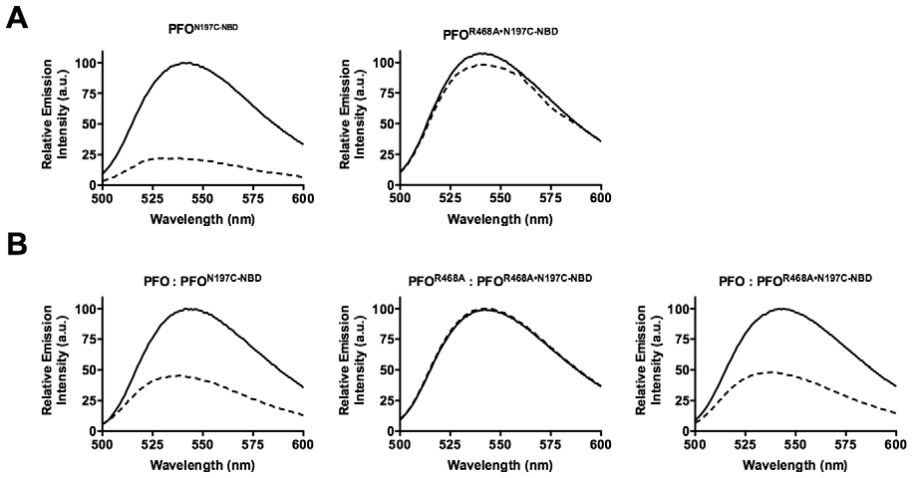
Disruption of the D2/D3 interface in PFO and PFO^R468A^. (A) A cysteine was substituted for TMH1 residue Asn-197, which is located within the D2/D3 interface. Each derivative was labeled with NBD and incubated in the presence (dashed line) and absence (solid line) of human erythrocyte ghost membranes. If TMH1 breaks its contact with D2 then Asn-197 moves from a buried, nonpolar location at the interface with D2 to the lumen of the pore. An NBD positioned at this location will therefore undergo a nonpolar to polar transition, which results in the quenching of the fluorescence emission. (B) Unlabeled native PFO or PFO^R468A^ were mixed at a 4∶1 molar ratio with PFO^N197C-NBD^ and PFO^R468A•N197C-NBD^ derivatives. The fluorescence emission intensity of NBD was measured from 500 to 600 nm. These data are representative of 3 experiments.

**Figure 6:**
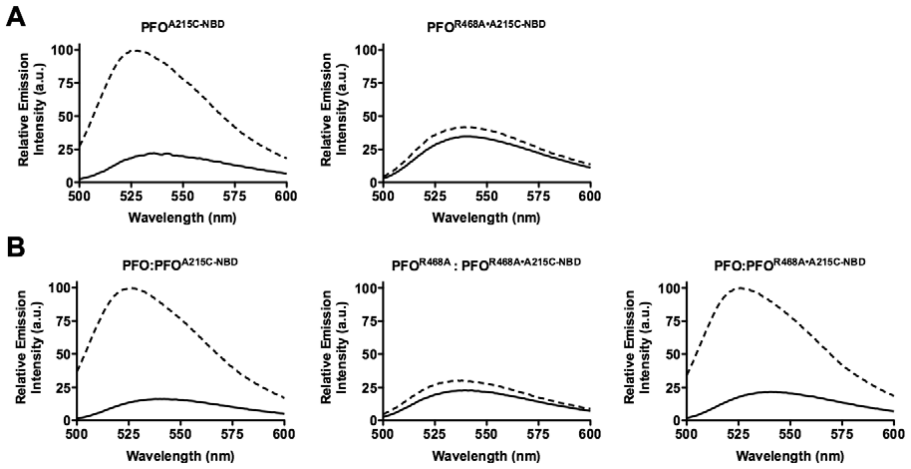
TMH insertion in PFO and PFO^R468A^. A cysteine was substituted for Ala-215 in PFO and PFO^R468A^, which is located in TMH1.The sidechain of Ala-215 is in an aqueous environment in the soluble monomer, but enters the membrane upon formation of the membrane spanning β-barrel [27]. Therefore an NBD probe positioned at this site undergoes a polar to nonpolar transition that is detected by an increase in the fluorescence emission of the probe.(A) Each NBD-labeled derivative was incubated in the presence (dashed line) and absence (solid line) of human erythrocyte ghost membranes. (B) Unlabeled native PFO or PFO^R468A^ were mixed in a 4∶1 molar ratio with PFO^A215C-NBD^ and PFO^R468A•A215C-NBD^ derivatives. The fluorescence emission for all experiments wasrelative to the maximum emission change observed for NBD-labeled PFO. The fluorescence emission intensity of NBD was measured from 500–600 nm. The data are representative of 3 experiments.

As expected, the fluorescence emission of the NBD probe on cysteine substituted Asn-197 in native PFO decreases to less than 25% of its initial value as the α-helical bundle disengages from its interface with D2 (Fig. 5A, left panel). Also, as expected, the fluorescence emission of the NBD probe located at position 215 in TMH1 of PFO increases as it makes the transition from its polar environment in the soluble monomer to its membrane embedded position in the β-barrel pore (Fig. 6A, left panel). In contrast, little change was detected in the fluorescence emission of the NBD probe at both locations in membrane bound PFO^R468A•N197C-NBD^, showing that TMH1 did not disengage from its interface with D2 (Fig. 5A, right panel) and insert into the membrane (Fig. 6A, right panel).

### Native PFO drives the membrane insertion of the PFO^R468A^ β-barrel pore

As shown above, the membrane-bound monomers of PFO^R468A^ do not interact and the D3 structural transitions that lead to the insertion of the β-barrel pore do not occur in PFO^R468A^: in essence the monomers remain inert after binding. Therefore, we next determined whether functional PFO could form chimeric oligomers with PFO^R468A^and drive these structural transitions.

The same experiments were performed as in Figs. 5A and 6A except that a 4∶1 ratio of unlabeled PFO or PFO^R468A^ was mixed with the labeled species prior to their addition to the liposomes. As expected, the relative emission intensity of the NBD probe was similar when each fluorescence species was mixed with a 4-molar excess of the unlabeled homologous protein. For PFO^N197C-NBD^ compare the left panels of Fig. 5A and Fig. 5B and for PFO^A215C-NBD^ compare the left panels of Fig. 6A and Fig. 6B. Similarly, no change was observed in the NBD emission for PFO^R468A•N197C-NBD^ (compare Fig. 5A, right panel to Fig. 5B center panel) and for PFO^R468A•A215C-NBD^ (compare Fig. 6A, right panel to Fig. 6B center panel) when they were mixed with a 4-molar excess of unlabeled PFO^R468A^.

However, when a 4-molar excess of unlabeled PFO was mixed with the NBD-labeled species of PFO^R468A^ it drove the disruption of the D2–D3 interface and insertion of the β-barrel pore. We observed the expected decrease in the fluorescence emission of the NBD probe located at the D2–D3 interface (compare the right and left panel of Fig. 5), as β5 swings away form β4. Also, the relative emission intensity increased as the probe located at position 215 inserted into the bilayer (compare the right and left panels in Fig. 6). Furthermore, the change in the emission intensity of the NBDin PFO^R468A•A215C-NBD^ when mixed with a 4-molar excess of PFO was quantitatively similar to that observed for PFO^A215C-NBD^ alone or mixed with the unlabeled PFO. Therefore, nearly all of the PFO^R468A•A215C-NBD^ TMHs were converted to a membrane-inserted state.

These results show that functional PFO can form sufficient intermolecular contacts with PFO^R468A^ to efficiently drive the disruption of its domain 2–3 interface and the membrane insertion of its β-barrel. Hence, PFO^R468A^is competent to undergo the necessary D3 structural changes and insert its TMHs into the membrane, but is unable to initiate these changes because it is missing the allosteric signal that is initiated by membrane binding. These data also indicate that the rate of binding of the PFO^R468A^ monomers to the membrane surface is not significantly different from that of the native PFO monomers, otherwise the PFO monomers would preferentially interact with each other before interacting with PFO^R468A^, which would have resulted in a less efficient conversion of the PFO^R468A^ monomers to an inserted state.

### Structural features of cholesterol-bound ILY

Our previous studies suggested that cholesterol binding by ILY was not necessary to trigger the D3 structural changes that are necessary for the formation of the oligomeric complex [32]: it appeared that CD59 binding, not cholesterol binding, initiated the D3 structural changes. Subsequent studies showed that the ILY CRM must initiate a cholesterol-dependent interaction to trigger the membrane insertion of loops L1–L3, which is necessary to anchor ILY to the membrane when it disengages from CD59 during prepore to pore conversion [33]. Therefore, if ILY could bind directly to cholesterol, in the absence of CD59, we predict that this interaction alone would not trigger the formation of the pore complex, as control of this process has been transferred to the CD59-binding site [30].

**Figure 7:**
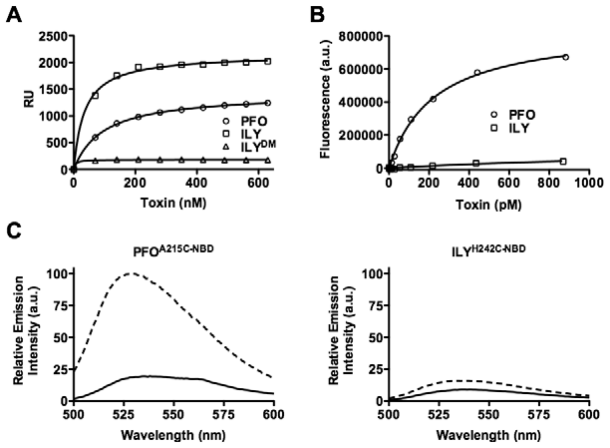
ILY binding and pore formation on cholesterol-rich liposomes. (A) Binding of PFO, ILY and ILY^DM^ to cholesterol-rich POPC liposomes was measured by SPR. The data is representative of 3 experiments. (B) Pore formation on liposomes was measured as the emission intensity of CF increased upon dilution as pores are formed in the liposomes. The change in the emission intensity of CF over time in an untreated sample was subtracted from the experimental data. The data are representative of at least 3 analyses. ILY^DM^ contains glycine substitutions for the ILY CRM residues Thr-517 and Leu-518, which knocks out CRM-dependent binding to cholesterol-rich membranes [22].(C) To measure the insertion of the β-barrel pore a cysteine was substitutedand modified with NBDfor TMH1 residue Ala-215 of PFO or its analog, His-242 in ILY. Each derivative was incubated in the presence (dashed line) and absence (solid line) of cholesterol-rich liposomes. As the soluble monomer binds to and forms a pore in the membrane the NBD probe positioned in TMH1 makes the transition from a polar environment in the soluble monomer (solid line) to the nonpolar environment of the membrane (dashed line), which is reflected by an increase in the NBD fluorescence emission [27].

Although ILY does not bind significantly to cholesterol-rich cell membranes that lack human CD59 [29], we unexpectedly discovered that it binds well to cholesterol-rich liposomes, even better than PFO (Fig. 7A). Furthermore, this binding is dependent on the CRM, as a CRM knockout (ILY^DM^) lacksdetectable binding to liposomes (Fig. 7A). Therefore, does this CRM-mediated binding trigger the D3 structural changes like PFO and formation of a β-barrel pore? To address this question we first generated cholesterol-rich liposomes with entrapped 5(6)-carboxyfluorescein (CF) and then treated them with PFO or ILY. The fluorescence emission of the concentrated liposome-trapped dye is quenched, but if the dye is released from the liposome its fluorescence emission increases upon dilution as it is released from the liposome [39], [40], [41]. PFO exhibited a dose-dependent release of the dye as evidenced by the increased emission of the dye as the concentration of PFO was increased. Although about twice as much ILY as PFO is bound to the liposomes, the ILY released less than 6% of dye released by PFO at the highest concentrations (Fig. 7B). We confirmed that the β-barrel of ILY was not inserting by measuring the insertion of TMH1. A NBD probe was position in TMH1 at cysteine-substituted His-242, which is a membrane facing residue in the β-barrel [42].Consistent with the lack of pore formation, ILY did not insert its β-barrel into the liposomal membranes (Fig. 7C).

**Figure 8:**
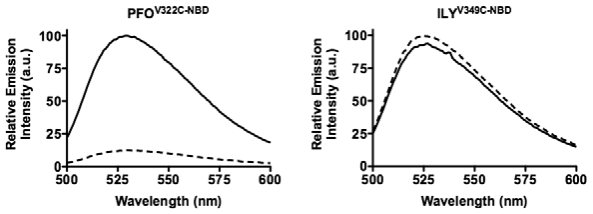
The β4–β5 interaction in cholesterol bound ILY. Cysteines were substituted for Val-322 in β4 of PFO or the analogous Val-349 in β4 of ILY and modified with NBD. Upon membrane binding β5 rotates away from β4, thus, the probe makes a transition from a nonpolar environment in the soluble monomer (solid line) to a polar environment in the membrane oligomer (dashed line), which results in the quenching of the NBD fluorescence [25]. The data are representative of 3 experiments.

Although pores were not forming, it was possible that the D3 β4–β5 interactionwas disrupted upon ILY binding to cholesterol-rich liposomes. The disruption of the β4–β5 interaction is detected by a decrease in the emission intensity of an NBD probe positioned in β4 as β5 rotates away from β4 its exposes the probe to the aqueous milieu thereby quenching its emission. This transition did not occur in the liposome bound ILY (Fig. 8), although it does occur in ILY bound to human CD59 containing cell membranes [32].Collectively these results suggest that while ILY can bind to cholesterol-rich POPC liposomes, like PFO^R468A^ its binding does not trigger the D3 structural transitions necessary to initiate the formation of the oligomeric pore complex.

## Discussion

The PFO pore forming mechanism is highly sensitive to changes in the undecapeptide structure [6], [13], [16], but until now the molecular basis for its role in the CDC mechanism has been elusive. The studies herein show that mutation of the undecapeptide arginine residue uncouples membrane binding from the D3 structural transitions, which are necessary for the assembly of the pore complex.This mutation blocks all detectable structural changes in D3and prevents the stable interaction of the membrane-bound monomers. In essence, the structure of membrane bound monomers of this mutant appears relatively unchanged from that of the soluble monomer. Hence, for the first time these studies demonstrate a function for the conserved undecapeptide, which forms a critical structural elementin the allosteric pathway that couples membrane binding to the D3 structural changes that lead to pore formation. The studies also show that binding initiates changes through this allosteric pathway that allow monomer-monomer interaction, but it is the monomer-monomer interactions that subsequently drive the major D3 structural transitions that are required for formation of the oligomeric pore complex.Furthermore, these studies show that this pathway has been lost in a CD59-binding CDC, which was a necessary evolutionary step towards transferring control of this process from the cholesterol-binding site to the CD59-binding site.

These studies show that mutation of Arg-468 disrupts the allosteric signal that couples binding to the D3 structural changes that lead to the formation of the oligomeric pore complex and that functional derivatives of PFO can drive the major structural changes in D3, which are necessary for membrane insertion of the TMHs of PFO^R468A^. Hotze et al. showed that monomer-monomer contact could drive the major structural transitions in D3 in a mutant of PFO that was trapped in a prepore complex. Here we show that PFO can drive these changes in PFO^R468A^, which is trapped at a much earlier stage where the monomers cannot interact with each other. Our data suggest that membrane binding is allosterically coupled to structural changes in PFO, which facilitate monomer-monomer interaction, but alone this allosteric pathway does not drive the major D3 structural transitions (i.e., disengagement of D2–3 interface and the β4–β5 interaction): these changes are driven by the subsequent interaction of monomers.

Soluble monomers of PFO do not interact and form oligomers, even at the high concentrations required for crystallization [7], [28]. Therefore, PFO membrane binding must initiate structural changes in the monomers that facilitate their interaction. The monomer-monomer interactions then drive the major conformational changes within domain 3, whichare required for the formation of the β-barrel pore [35]. In this way PFO ensures an efficient assembly of the oligomeric pore complex on the target membrane. Membrane-bound PFO^R468A^ monomers did not appear to form interactions that were of sufficient duration to be detected by FRET. For FRET to occur the donor and acceptor fluorophores must be at a fixed distance for a time that is equal to or greater than the half-life of the donor fluorescence emission, which is approximately 4 nsec for the Alexa-488 dye [38]. Therefore, the mutation of Arg-468 appears to prevent the changes in the monomer structure that allows monomers to initially interact and form stable contacts that then drive the D3 structural changes. This mutation results in a membrane-bound monomer that appears to retain the structure of the soluble monomer, which cannot form any detectable intermolecular interactions.

In the crystal structure of PFO the only contacts made by Arg-468 are hydrogen bonds between the NH1 of its guanidinium group and the backbone carbonyls of the CRM Thr-Leu pair (Fig. 1). Therefore, its substitution with alanine only prevents the formation of these two hydrogen bonds. This contact is interesting because it hydrogen bonds with the CRM, and may help stabilize the CRM structure in PFO. Hence, this contact may explain why mutation or chemical modification of the undecapeptide affects binding, as well as assembly of the oligomeric pore complex of PFO [6], [13], [16]. We cannot know with certainty, however, that this contact is essential to the allosteric pathway, only that the substitution of Arg-468 disrupts the allosteric pathway that couples membrane binding to the formation of the pore complex. The crystal structures of the cholesterol-binding CDCs PFO [7], suilysin (SLY) [43] and anthrolysin O (ALO) [9] have revealed that the undecapeptide 3D structure is highly variable: no two undecapeptides 3D structures have been shown to be the same [7], [9], [43], even though their undecapeptide primary structures are identical. It is possible that these differences are due to an inherent flexibility of the undecapeptide and/or crystal contacts that affect the structural arrangement of the undecapeptides in the crystals. Hence, the conformational coupling of binding to the D3 structural changes may proceed through different undecapeptide mediated contacts in the CDCs. Alternatively, if the structure of the undecapeptide is flexible, as is suggested by the crystal structures, then membrane binding may lock it into a specific conformation that transmits the allosteric signal to D3, which cannot be achieved in PFO^A468A^.

Functional PFO can drive D3 conformational changes in PFO^R468A^and the membrane insertion of itsTMHs in chimeric oligomers comprised of both proteins. Therefore, PFO^R468A^ is structurally competent to form a pore, but lacks the conformational signal that initiates the necessary changes in its structure that facilitate the formation of stable intermolecular contacts. The fact that native PFO can drive these structural changes in PFO^R468A^ indicates that it can establish a sufficient number of contacts with the PFO^R468A^ monomers to drive these conformational changes in the latter. No stable intermolecular interactions of the PFO^R468A^ monomers alone were detected by FRET showing that they do not interact, or that the interactions are transient and only exist on a timescale that cannot be detected by FRET. The ability of functional PFO derivatives to interact with PFO^R468A^ indicates that at least one of the surfaces of PFO^R468A^is accessible to the functional PFO derivatives, which allows the functional PFO derivatives to dock with PFO^R468A^.This interaction allows the functional PFO derivatives to establish contact with andsubsequently drive the structural changes in PFO^R468A^D3 that are necessary for the formation of the oligomeric pore complex.

It is clear thatPFO^R468A^bindingwas also affected by the Arg-468 to alanine mutation. If Arg-468 does make contact with the CRM carbonyls, as suggested by the PFO crystal structure, then it is possible that this substitution partially destabilized the CRM structure thereby affected binding. However, avidity may be a more important factor that contributes tothe difference in binding of wildtype PFO and PFO^R468A^. Wildtype PFO and all mutants thereof generated to date still form membrane oligomers (most are represented herein). Oligomerizationis an important component of the binding interaction due to the avidity of the oligomeric complex versus the binding affinity of a single monomer. Oligomerization of PFO begins shortlyafter binding [37], [44], thus binding of wildtype PFO and its derivatives reflects the avidity of the oligomeric complex rather than the affinity of single monomer.PFO^R468A^ is the first mutant that has been described, whichis trapped in a monomer state on the membrane.Hence, therelatively poorbinding exhibited by PFO^R468A^ may actually reflect the true binding interaction of native PFO monomers in the absence of oligomerization. It is also important to note that in the experiments in which functional PFO was used to drive the structural transitions in PFO^R468A^ that we obtained near quantitative conversion of these transitions with a 4∶1 molar ratio of functional PFO to PFO^R468A^. If PFO^R468A^ monomers bound the membrane with a significantly lower affinity than native PFO monomers then the probability of the native PFO monomers interacting with the PFO^R468A^ monomers would be decreased and therefore it would be unlikely that we would have observed the near quantitative conversion of the PFO^R468A^ monomers to a membrane-embedded state.

The CD59-binding CDCs, ILY [29], vaginolysin (VLY) [31] and lectinolysin (LLY) [45], [46] exhibit undecapeptides with significant changes to their primary structures, most notably a proline substitution for the second conserved tryptophan (consensus, ECTGLAWEWWR;ILY, GATGLAWEPWR; VLY, EKTGLVWEPWR; LLY, EKTGLVWEPWR). Unlike PFO, ILY does not maintain the hydrogen bond contacts between Arg-495 and the CRM (Fig. 1). This may be one of contacts in the cholesterol-dependent allosteric pathway that was disrupted during the evolution of the CD59-binding site, which was necessary to transfer of control of the D3 structural changes from the cholesterol-binding site to the CD59-binding site. Consistent with this scenario is the observation that substitution of the analogous arginine residue in ILY has little effect on the ILY pore-forming mechanism [6]. We have shown herein that when ILY binds to cholesterol in the absence of CD59 it remains largely inert on the membrane, similar to what we observed for PFO^R468A^. These data suggest that through evolution ILY has lost the allosteric pathway that couples cholesterol binding to the D3 structural changes in order to transfer control of the assembly of the oligomeric complex to the CD59-binding site [33], [42], [46].

Recently others have proposed that the membrane attack complex/perforin (MACPF) family of proteins may exhibit a CDC-like pore forming mechanism [47], [48], [49], [50], [51]. This proposal is based on the presence of a conserved protein fold that is similar to D3 of the CDCs [7], which we have shown forms the β-barrel pore structure of the CDCs [26], [27]. The MACPF proteins play important roles in immune defense as well as in the pathogenesis of eukaryotic pathogens such as *Toxoplasma gondii* [52]and *Plasmodium falciparum* [53], [54], [55]. These proteins exhibit little sequence homology with the CDCs and do not exhibit an undecapeptide motif. It is possible, however, that they will alsoexhibit an analogous allosteric mechanism to regulate the assembly of their pore complex.

These studies provide the first evidence that shows the conserved undecapeptide plays an integral role in the allosteric coupling of cholesterol-mediated membrane binding to distal structural changes, which are necessary for the monomer-monomer interactions that drive the assembly of the β-barrel pore.

## Materials and Methods

### Bacterial strains, plasmids and chemicals

The genes for native ILY and PFO were cloned into pTrcHisA (Invitrogen) as described previously [27], [42]. All mutations were made in native ILY (naturally cysteine-less) or the cysteine-less PFO derivative (PFO^C459A^) backgrounds. The various CDCs and their derivatives are summarized in Table 2. All chemicals and enzymes were obtained from Sigma, VWR and Research Organics. All fluorescent probes were obtained from Molecular Probes (Invitrogen). Polyclonal anti-PFO antibody was affinity purified from hyperimmune rabbit serum. Secondary antibody goat anti-rabbit-HRP was obtained from BioRad. Sterols were obtained from Steraloids and lipids were obtained from Avanti Polar Lipids.

**Table 2:** PFO and its derivatives used herein.

Toxin or its derivative	Description
PFO	Recombinant native PFO that contains a alanine substitution for the native cysteine (Cys-459) in the undecapeptide [27]
PFO^R468A^	PFO substituted at Arg-468 with alanine
PFO^β4β5^	PFO with an engineered disulfide between β-strands 4 and 5 at cysteine substituted Thr-319 in β4 and Val-334 in β5
PFO^R468A•β4β5)^	The analogous mutation to PFO^β4β5^
PFO^V322C^	PFO with an engineered cysteine for Val-322. Val-322 is located in β4 and is buried under the α1β5 loop. It undergoes a nonpolar to polar transition as the α1β5 loop rotates away from β4 [25]
PFO^R468A•V322C^	The analogous mutation toPFO^V322C^
PFO^N197C^	Cysteine-substituted Asn-197. Asn-197 undergoes a nonpolar to polar transition upon disruption of the D2–D3 interface [27]
PFO^R468A•N197C^	The analogous mutation toPFO^N197^
PFO^A215C^	Cysteine-substituted Ala-215 in TMH1. Ala-215 faces the membrane in the β-barrel pore [27]
PFO^R468A•A215C^	The analogous mutation to PFO^A215C^
ILY	Recombinant native ILY
ILY^DM^	Cholesterol binding site knockout by glycine substitution for the Thr-Leu pair of the CRM in ILY
ILY^H242C^	The analogous mutation in ILY to PFO^A215C^
ILY^V349C^	The analogous mutation in ILY to PFO^V322C^

A summary of the CDCs and their derivatives used in the present study is shown.

### Generation and purification of toxin derivatives

PCR QuikChange mutagenesis (Stratagene) was used to make the various amino acid substitutions in native ILY or PFO^C459A^ and DNA sequences of the PFO and ILY mutants were determined by the Oklahoma Medical Research Foundation Core DNA Sequencing Facility. The expression and purification of recombinant toxins and derivatives in*Escherichia coli*BL21 DE3 were carried out as previously described [27], [56]. Purified protein was stored in HBS [100 nM NaCl, 50 mM HEPES; (pH 7.5)], 50 µMtris(2-carboxyethyl)phosphine (TCEP) and 10% (vol/vol) glycerol at −80°C.

### Modification of cysteine-substituted toxin derivatives with fluorescent probes

The labeling of PFO, PFO^R468A^ and ILY cysteine-containing derivatives with IANBD [iodoacetamido-N,N′-dimethyl-N-)7-nitrobenz-2-oxa-1,3-diazolyl)ethylene-diamine; Molecular Probes] was carried out as previously described [27], [42]. Toxin derivatives were labeled using a 20-fold molar excess of the probe overnight at room temperature (22°C).The labeling reactions for the PFO^V322C^ derivatives also contained 3 M guanidine hydrochloride to increase the efficiency of labeling.

Following the modification with the probes the mixtures were passed over a Sephadex G-50 column equilibrated in HBS. The labeled samples were made 10% (vol/vol)in glycerol and stored at −80°C. Proteins were typically labeled at an efficiency of 80–100%.

### Hemolytic activity of toxins and derivatives

The cytolytic activity of the toxins and their derivatives on human red blood cells (hRBCs) was measured as previously described [27] except that the procedure was adapted to a microtiter plate format. Briefly, fresh human RBCs (hRBCs) were washed and suspended to 5% in phosphate buffered saline (PBS). The PFO and its derivatives were serially diluted in 2-fold steps in a microtiter plate at a final volume of 50 µl per well to which 50 µl of a 5% suspension of hRBCs was added and incubated for 1 hour at 37°C. After incubation, unlysed RBCs were removed by centrifugation of the plate at 3400×g for 10 min. The EC_50_for hemolysis (effective concentration of toxin for 50% hemolysis)was determined by quantifying hemoglobin release by measuring the absorbance of the supernatantat 540 nm using a DU640B spectrophotometer (Beckman).

### Liposome preparation

Liposomes containing 1-palmitoyl-2-oleoyl-sn-glycero-3-phophocholine (POPC) and cholesterol at a ratio of 45∶55 mol% were prepared as previously described [27]. Carboxyfluorescein-containing liposomes were made by adding CF [5(6)-carboxyfluorescein]to the cholesterol/lipid mixture in HBS at a concentration of 50 mM before extruding [40]. After extrusion, the encapsulated liposomes were then passed over a Sephadex G-50 column in HBS pH 7.5 to separate unencapsulated CF from liposomes.

### Surface plasmon resonance analysis of ILY and PFO liposome binding

Surface plasmon resonance (SPR) was performed with a BIAcore 3000 system (Oklahoma Medical Research Foundation) using an L1 sensor chip (Biacore) as previously described [22]. Binding analysis was performed as previously described [22] with the following modification: nine consecutive 10 µl injections of the toxins and their derivatives (100 ng per injection) in HBS were passed over the liposome-coated chip at a flow rate of 10 µl/min.

### Liposome release assay

The pore forming activity of PFO and ILY and ILY DM was measured by incubating serial dilutions of toxin with 100 µl of a 1∶1000 dilution of carboxyfluorescein (CF)-containing liposomes in HBS for 1 h at 37°C. Samples were read on a Victor3V Wallac 1420 Multilabel counter (Perkin Elmer) using wavelength settings optimized for high count fluorescein detection.

### Flow cytometry

Two-fold serial dilutions of PFO^β4β5^and PFO^R468A•β4β5^labeled with Alexa-488 were incubated with washed human RBCs (1×10^6^ cells) in PBS for 30 min at 4°C.Samples were then brought to a final volume of 500 µl with cold PBS and analyzed by a FACSCalibur flow cytometer (University of Oklahoma Health Sciences Center), gating on live cells. The emission wavelength was 530 nm and the excitation was 488 nm with a bandpass of 30 nm. The disulfide locked β4β5 versions of PFO and PFO^R468A^ have cysteines substituted for residues Thr-319 and Val-334 [25], which forms a disulfide that prevents β5 from rotating away from β4. This disulfide prevents the lysis, but not binding to the RBCs during flow cytometry [25].

The geometric mean fluorescence of RBCs alone was subtracted from the experimental data for both PFO derivatives and the net fluorescence was graphed using GraphPad Prism.

### SDS-agarose gel separation of PFO monomer and oligomers

SDS-agarose gel electrophoresis was performed as previously described [36]. Briefly, samples were incubated with different concentrations of washed hRBCs, for 30 min at 37°C.Toxins were maintained at 440 nM and the hRBCs concentrations ranged from 2.5×10^7^/ml to 2.5×10^8^/ml in a final volume of 40 µl. Samples were solubilized with SDS sample buffer and the complexes were analyzed on a 1.5% SDS-agarose gel (100 V, 120 min) and then transferred to nitrocellulose membranes. Protein bands were identified using rabbit anti-PFO antibody followed by horseradish peroxidase tagged goat anti-rabbit secondary IgG. The bands were visualized using a chemiluminescent substrate (ECL Western Blotting Detection Reagents, Amersham/GE Healthcare) and autoradiography.

### Human erythrocyte ghost membrane preparation

Human erythrocyte (hRBC) ghost membranes were prepared as previously described with some modifications [27], [42]. After hypotonic lysis of the hRBCs for 15 min at 4°C in lysis buffer [5 mM sodium phosphate (monobasic), pH 7.5, 1 mM EDTA], cytoplasmic constituents were separated from the membranes by dialysis with 2 L of the lysis buffer by recirculation through a Vivaflow 200 0.2 µm PES cassette (Sartorius Stedim Biotech). Membrane protein content was quantified using the Bradford method (Bio-Rad Protein Assay, Bio-Rad Laboratories, Inc.) as previously described [27].

### Fluorescence spectroscopy

All fluorescence intensity measurements were performed using a Fluorolog-3 Spectrofluorometer with the fluorescence software (Horiba JobinYvon). NBD measurements were made using the following settings: an excitation wavelength of 480 nm and an emission wavelength of 540 nm with a bandpass of 5 nm. Emission intensity was scanned between 500 and 600 nm at a resolution of 1 nm with an integration time of 0.1 sec. In a typical experiment, labeled and unlabeled samples containing 10 µg total toxin each were incubated with hRBC ghost membranes (equivalent to 300 µg of membrane protein) or 20 µl liposomes in HBS for 15 min at 37°C before taking spectral measurements. For all experiments the fluorescence intensity of the unlabeled samples was subtracted from that of the fluorescent probe-labeled samples in order to control for the intrinsic fluorescence of the sample in the absence of the probe.

### Fluorescence resonance energy transfer (FRET) measurements

FRET analysis was performed as previously described [35] with the following changes. The PFO and PFO^R468A^ derivatives were labeled with either Alexa Fluor 488 (donor, D) or Alexa Fluor 568 (acceptor, A).Parallel samples were prepared containing 10 µg of D-labeled toxin mixed with a 4-fold molar excess of either A-labeled toxin or unlabeled (U) toxin in a total volume of 2 ml. To correct for light scattering and direct excitation of the acceptor, a sample was prepared in parallel in which unlabeled PFO or PFO^R468A^ (U) replaced the donor-labeled PFO to create the UA sample, therefore net DA = DA-UA. The samples were mixed in the presence of hRBC ghost membranes (equivalent to 300 µg of membrane protein) for 15 minutes at 37°C and the donor emission intensity measured from 500 nm to 600 nm. The donor emission intensity of samples in which unlabeled PFO derivatives replaced donor-labeled PFO derivatives was measured and subtracted from the donor-labeled samples to control for any intrinsic fluorescence of the toxin or direct excitation of the acceptor.
