# Correction: The Cholesterol-Dependent Cytolysin Signature Motif: A Critical Element in the Allosteric Pathway that Couples Membrane Binding to Pore Assembly

**DOI:** 10.1371/annotation/750e7055-3a67-44ac-88e1-673d017a15c7

**Published:** 2012-08-08

**Authors:** Kelley J. Dowd, Rodney K. Tweten

Dr. Allison J. Farrand was not included in the author byline. She should be listed as the second author and affiliated with Department of Microbiology and Immunology, The University of Oklahoma Health Sciences Center, Oklahoma City, Oklahoma, United States of America. The contribution of this author is as follows: She constructed and qualified the basic features of several of the mutants of PFO and ILY used in the published studies.

